# Comparison of 3 diagnostic methods for pulmonary tuberculosis in suspected patients with negative sputum smear or no sputum

**DOI:** 10.1097/MD.0000000000037039

**Published:** 2024-02-09

**Authors:** Xiaopeng Cheng, Lerong Chen, Wenli Wan, Jianping Peng, Liangliang Wu, Jing Xin, Jianying Cai

**Affiliations:** a Department of Respiratory and Critical Care Medicine, Jiangxi Chest Hospital, Nanchang City, Jiangxi Province, China; b Disinfection supply center, Jiangxi Chest Hospital, Nanchang City, Jiangxi Province, China.

**Keywords:** bronchoalveolar lavage fluid, diagnostic assays, nucleic acid amplification testing, pulmonary tuberculosis, sputum, sputum smear

## Abstract

**Study design:**

To explore the diagnostic value of 3 methods for sputum smear-negative and non-sputum patients with suspected pulmonary tuberculosis (TB).

**Methods:**

This prospective study enrolled sputum smear-negative and non-sputum patients with suspected TB admitted to Jiangxi Chest Hospital between January 2020 and December 2022. The 3 methods were bronchoalveolar lavage fluid (BALF)-acid-fast bacillus (AFB) smear, GeneXpert MTB/RIF, and gene chip for *Mycobacterium* strain identification. The diagnostic performance of the 3 tests was evaluated with BALF Mycobacterium culture + BALF-AFB smear + GeneXpert MTB/RIF + Gene chip as the gold standard.

**Results:**

A total of 456 samples were collected from 114 patients with suspected TB. Twenty-four patients were diagnosed with TB. The combination of GeneXpert MTB/RIF and gene chip for *Mycobacterium* strain identification yielded the highest area under the receiver operating characteristics curve (AUC) of 0.953 and had sensitivity of 90.57%, specificity of 100%, positive predictive value (PPV) of 100%, negative predictive value (NPV) of 92.42%, accuracy of 95.61%. GeneXpert MTB/RIF achieved AUC of 0.906, sensitivity of 81.13%, specificity of 100%, PPV of 100%, NPV of 85.92%, accuracy of 91.23%. BALF-AFB smear had AUC of 0.519, sensitivity of 3.77%, specificity of 100%, PPV of 100%, NPV of 54.46%, and accuracy of 55.26%. The combination of GeneXpert MTB/RIF and gene chip for *Mycobacterium* strain identification yielded the highest κ of 0.911, while BALF-AFB smear had the lowest κ value of 0.040.

**Conclusion:**

For TB in sputum smear-negative and non-sputum patients using BALF Mycobacterium culture + BALF-AFB smear + GeneXpert MTB/RIF + Gene chip as the gold standard, BALF-AFB smear showed low diagnostic performance, while, though GeneXpert MTB/RIF and gene chip had good diagnostic performance, combining GeneXpert MTB/RIF and gene chip improved the diagnostic value to a great extent.

## 1. Introduction

Pulmonary tuberculosis (TB) refers to the clinical syndrome associated with respiratory system infection caused by *Mycobacterium tuberculosis*.^[1]^ The World Health Organization estimated that 10 million people would develop TB and 1.5 million would die from the disease in 2018, making TB a global crisis, especially under the circumstance of antibiotic resistance.^[2]^ *M. tuberculosis* is spread through the air from 1 person to another when bacteria are aerosolized from a person with pulmonary TB.^[3,4]^

TB should be suspected in patients with suggestive symptoms, including productive cough for over 2 weeks, dyspnea, chest pain, hemoptysis, loss of appetite, weight loss, fever, and night sweats, with history of exposure or residence in endemic locations.^[1,5]^ The identification of *M. tuberculosis* in respiratory specimens is used to confirm the diagnosis of pulmonary TB in patients with compatible clinical symptoms.^[1,5]^ The tests used for bacteriologic diagnosis include acid-fast bacillus (AFB) smear microscopy, which is not specific to *M. tuberculosis*, nucleic acid amplification testing (NAAT), including the *M. tuberculosis* Direct (MTD) test and the GeneXpert MTB/Rif which also tests for rifampicin susceptibility, gene chips, as well as liquid and solid mycobacterial culture (i.e., the gold standard for diagnosis). The GeneXpert MTB/Rif and gene chip assays were reported to be more sensitive than culture for detecting *M. tuberculosis* in sputum.^[6]^ The GeneXpert MTB/Rif assay also tended to be more accurate than AFB.^[7]^

Still, culture-negative, sputum smear-negative pulmonary TB should be considered in patients whom an adequate workup had been already conducted in^[8,9]^ since such patients might represent 30% to 60% of the patients with TB.^[10]^ Indeed, failure to isolate *M. tuberculosis* from appropriately collected samples could not exclude a diagnosis of active TB in patients with positive clinical or radiographic findings.^[8,9]^ Before making a definite diagnosis, bronchoscopy with bronchoalveolar lavage fluid (BALF) and biopsy should be considered.^[10]^ Of note, sputum smear-negative TB is likely to be underdiagnosed and can contribute to the widespread of TB in some areas,^[11]^ especially since such patients can present with no cough or radiologic signs.^[12]^ Effective diagnostic methods are necessary to avoid underdiagnosis and empirically treat patients without TB.^[12]^ A study suggested that the GeneXpert MTB/Rif assay and culture had similar *M. tuberculosis* detection rates with faster result-producing speed in patients with smear microscopy-negative sputum specimens.^[13]^

Therefore, this study aimed to explore the diagnostic value of BALF-AFB smear, GeneXpert MTB/RIF, and gene chip for *Mycobacterium* strain identification in sputum smear-negative and non-sputum patients with suspected pulmonary TB.

## 2. Material and methods

### 2.1. Study design and patients

This prospective study enrolled sputum smear-negative and non-sputum patients with suspected TB admitted to the Respiratory and TB Departments of Jiangxi Chest Hospital between January 2020 and December 2022. The study was approved by the Medical Ethics Committee of Jiangxi Chest Hospital (#2019-42). All patients signed the informed consent.

The inclusion criteria were: ≥ 18 years of age; typical TB symptoms, including cough, chest pain, blood in sputum or hemoptysis, fever, night sweats, weight loss, and imaging manifestations, including patches, spots, and cavities, or history of contact with patients with TB; and negative sputum smear or no sputum.

The exclusion criteria were: other serious lung diseases, such as lung cancer, or interstitial lung disease; prior treatment with anti-TB drugs; surgery, chemotherapy, radiotherapy, or other treatments within 3 months; or contraindications to bronchoscopy, such as severe bleeding tendency, coagulation mechanism disorder, uncontrolled hypertension of blood pressure >160/100 mm Hg, anesthetic drug allergy, severe respiratory insufficiency, or consciousness disturbance.

### 2.2. Measurements

Bronchoalveolar lavage was performed every morning to collect BALF and sputum samples for BALF *Mycobacterium* culture as the gold standard, BALF-AFB smear, GeneXpert MTB/RIF, and gene chip for *Mycobacterium* strain identification.

#### 2.2.1. BALF-AFB smear.

According to the TB laboratory diagnostic technique guidelines approved by the Center for TB Prevention and Control of the Chinese Center for Disease Control and Prevention, a direct smear of each sputum or BALF sample was tested by BALF-AFB smear.

#### 2.2.2. BALF Mycobacterium culture.

The samples were digested with n-acetyl-L-cysteine (NALC) and NALC-NaOH for 15 minutes and purified with phosphate buffer saline. The samples were centrifuged at 3000 × *g* for 15 minutes. The supernatant was discarded, and the sediment was re-suspended with 1.5 mL of phosphate buffer saline. Then, 0.2 mL of suspension was seeded onto the surface of the Lowenstein-Jensen medium. Each petri dish was observed and recorded weekly until the growth of colonies was found. The growth of bacterial colonies was observed and recorded every week, and fresh colonies were selected for mycobacterium strain identification by MPT64 antigen kit. A sample was considered negative if no growth of colonies was found after 8 weeks.

#### 2.2.3. GeneXpert MTB/RIF.

The sample (1.0 mL) was mixed with 2.0 mL of GeneXpert MTB/RIF sample digestion reagent and incubated at room temperature for 15 minutes. Then, 2.0 mL of the digested sample was added to the GeneXpert MTB/RIF for analysis.

#### 2.2.4. Gene chip for mycobacterium strain identification.

First, 200 μL of reaction reagent A was added to the chip box window so that the surface of the chip film was fully soaked. After reagent A was fully infiltrated, the chip was placed at room temperature for 1 minute, and 100 μL of the sample was added. After the sample was completely infiltrated, 300 μL of reaction reagent B was added. After reagent B was completely infiltrated, 50 μL of reagent C was added. After reagent C was completely infiltrated, 300 μL of reagent D was added. The chips were put into the biochip recognition apparatus within 30 minutes after the completion of the reaction for analysis. The detailed procedures were showed in Supplement materials.

### 2.3. Outcome

The outcome was the diagnosis of TB based on typical TB symptoms, imaging features, negative etiology, effective TB therapy, no other diseases such as bacterial pneumonia, lung cancer, sarcoidosis, asthma, bronchiectasis, or non-TB mycobacterium infection, and positive result of BALF *Mycobacterium* culture according to “*People Republic of China Health Industry Standard Tuberculosis Diagnosis (WS 288-2017*).”

### 2.4. Data collection and follow-up

The baseline characteristics of the patients, including age, sex, complications, symptoms, and imaging features, were collected from the hospital electronic medical record system. From January 2020 to November 2022, the patients were followed up by outpatient visits or telephone once a month for a total of 6 months. Chest computed tomography, routine blood test, and blood biochemistry examinations were performed at each follow-up. During the telephone follow-up, the patient reported the results of examination from the local hospital.

### 2.5. Statistical analysis

SPSS 25.0 (IBM Corp., Armonk, NY, USA) was used for statistical analysis. The continuous data with skewed distribution were expressed as median (IQR) and analyzed using Mann–Whitney *U* test. The categorical data were expressed as n (%) and analyzed using the Pearson chi-square test. The 3 diagnostic methods were evaluated using the area under the receiver operating characteristics curve (AUC), sensitivity, specificity, positive predictive value (PPV), negative predictive value (NPV), accuracy, and Cohen kappa which was used to analysis κ value. The BALF Mycobacterium culture + BALF-AFB smear + GeneXpert MTB/RIF + Gene chip for Mycobacterium strain were identified as the gold standard. The McNemar test was used to analyze the paired data. Two-sided *P* values <.05 were considered statistically significant.

## 3. Results

### 3.1. Characteristics of the patients

**Figure 1.**
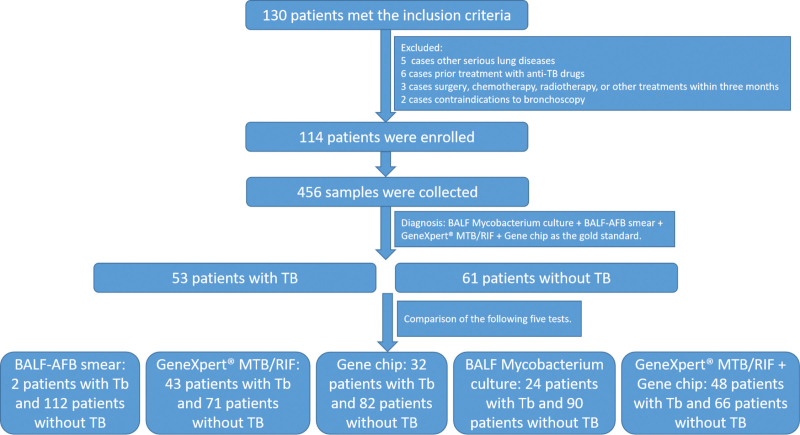
Study flowchart.

During the study period, 130 patients met the inclusion criteria, 16 were excluded, and 114 patients with suspected TB were ultimately enrolled. A total of 456 samples were collected from the 114 patients. Among them, 53 patients were diagnosed with TB according to BALF Mycobacterium culture + BALF-AFB smear + GeneXpert MTB/RIF + Gene chip, while 61 patients showed negative. The flowchart of this study were showed in Figure 1. Compared with the non-TB group, patients in the TB group were significantly younger [32.00 (21.00 53.50) vs 49.00 (32.50 54.50) years, *P* = .027]. There were no significant differences in sex distribution between the 2 groups (male: 67.92% vs 62.30%, *P* = .530). Table 1 presents the baseline characteristics of the patients.

**Table 1:** Baseline characteristics of the patients according to TB diagnosed according to BALF Mycobacterium culture + BALF-AFB smear + GeneXpert MTB/RIF + Gene chip.

Characteristics	TB (n = 53)	Non-TB (n = 61)	*P*
Age (yr)	32.00 (21.00 53.50)	49.00 (32.50 54.50)	.027
Sex, n (%)			.530
Male	36 (67.92)	38 (62.30)	
Female	17 (32.08)	23 (37.70)	
Complications, n (%)			
Hypertension	9 (16.98)	4 (6.56)	
Diabetes	2 (3.77)	2 (3.28)	
Coronary heart disease	1 (1.89)	0	
Mild COPD	1 (1.89)	2 (3.28)	
Bronchiectasis	1 (1.89)	1 (1.64)	
Hepatitis B	1 (1.89)	4 (6.56)	
Symptom, n (%)			
Fever	2 (3.77)	3 (4.92)	
Cough	15 (28.30)	20 (32.79)	
Expectoration	27 (50.94)	41 (67.21)	
Night sweats	20 (37.74)	14 (22.95)	
Sputum/hemoptysis	7 (13.21)	1 (1.64)	
Chest distress	11 (20.75)	9 (14.75)	
Chest pain	6 (11.32)	16 (26.23)	
Imaging features, n (%)			
Cavities shadow	8 (15.09)	14 (22.95)	
Patch infiltration shadow	27 (50.94)	30 (49.18)	
Fibrosclerosis	33 (62.26)	44 (72.13)	
Miliary shadow	11 (20.75)	19 (31.15)	
Nodular mass shadow	12 (60.00)	19 (59.38)	
Pleural effusion	2910.000	4 (12.50)	
Nodular mass shadow	13 (54.17)	53 (58.89)	
Pleural effusion	2 (8.33)	8 (8.89)	

COPD = chronic obstructive pulmonary disease, TB = tuberculosis.

### 3.2. Diagnostic value of the 3 methods for TB

**Figure 2.**
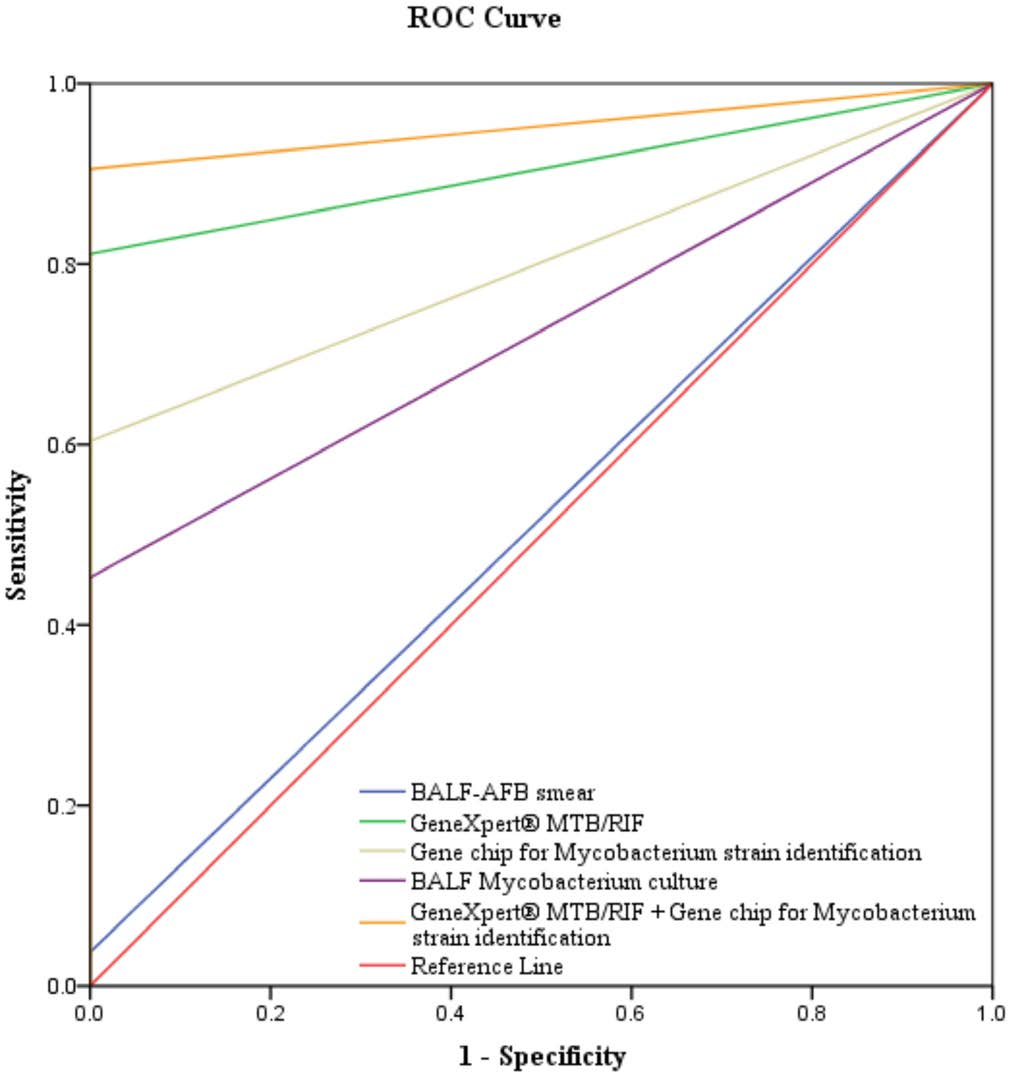
ROC curve for BALF-AFB smear, GeneXpert MTB/RIF, Gene chip for *Mycobacterium strain* identification, and GeneXpert MTB/RIF combined with Gene chip for *Mycobacterium strain* identification. AFB = acid-fast bacillus, BALF = bronchoalveolar lavage fluid.

In the present study, the combination of GeneXpert MTB/RIF and gene chip for *Mycobacterium* strain identification yielded the highest AUC of 0.953 (Fig. 2) and had sensitivity of 90.57%, specificity of 100%, PPV of 100%, NPV of 92.42%, and accuracy of 95.61%. On the other hand, GeneXpert MTB/RIF achieved AUC of 0.906, sensitivity of 81.13%, specificity of 100%, PPV of 100%, NPV of 85.92%, accuracy of 91.23%. Gene chip for *Mycobacterium* strain identification achieved AUC of 0.802, sensitivity of 60.38%, specificity of 100%, PPV of 100%, NPV of 74.39%, accuracy of 81.58%. BALF-AFB smear had AUC of 0.519, sensitivity of 3.77%, specificity of 100%, PPV of 100%, NPV of 54.46%, and accuracy of 55.26% (Table 2).

**Table 2:** Diagnostic value of the 3 methods with BALF *Mycobacterium* culture + BALF-AFB smear + GeneXpert MTB/RIF + Gene chip for *Mycobacterium* strain identification as the gold standard.

Methods	AUC	Sensitivity	Specificity	PPV	NPV	Accuracy
BALF-AFB smear	0.519 (0.412–0.626)	3.77%	100%	100%	54.46%	55.26%
GeneXpert MTB/RIF	0.906 (0.841–0.970)	81.13%	100%	100%	85.92%	91.23%
Gene chip for *Mycobacterium* strain identification	0.802 (0.715–0.889)	60.38%	100%	100%	74.39%	81.58%
BALF *Mycobacterium* culture	0.726 (0.629–0.824)	45.28%	100%	100%	67.78%	74.56%
GeneXpert MTB/RIF + Gene chip for *Mycobacterium* strain identification	0.953 (0.906–1.000)	90.57%	100%	100%	92.42%	95.61%

AFB = avid-fast bacilli, BALF = bronchoalveolar lavage fluid, MTB = mycobacterium tuberculosis, NPV = negative predictive value, PPV = positive predictive value, RIF = rifampicin.

### 3.3. Agreement between the 3 diagnostic methods and BALF *Mycobacterium* culture

GeneXpert MTB/RIF had a κ of 0.821, and the gene chip for *Mycobacterium* strain identification had a κ of 0.620. The combination of GeneXpert MTB/RIF and gene chip for *Mycobacterium* strain identification yielded the highest κ of 0.911. BALF-AFB smear had the lowest κ value of 0.040, indicating poor agreement with BALF *Mycobacterium* culture as golden standard (Table 3).

**Table 3:** Test results of the 3 methods with BALF *Mycobacterium* culture + BALF-AFB smear + GeneXpert MTB/RIF + Gene chip for *Mycobacterium* strain identification as the gold standard.

Methods		Gold standard		Total	κ
		Positive	Negative		
BALF-AFB smear	Positive	2	0	2	0.040
	Negative	51	61	112	
GeneXpert MTB/RIF	Positive	43	0	43	0.821
	Negative	10	61	71	
Gene chip for *Mycobacterium* strain identification	Positive	32	0	32	0.620
	Negative	21	61	82	
BALF *Mycobacterium* culture	Positive	24	0	24	0.470
	Negative	29	61	90	
GeneXpert MTB/RIF + Gene chip for *Mycobacterium* strain identification	Positive	48	0	48	0.911
	Negative	5	61	66	

AFB = avid-fast bacilli, BALF = bronchoalveolar lavage fluid, MTB = mycobacterium tuberculosis, RIF = rifampicin.

## 4. Discussion

This study showed that, for TB in sputum smear-negative and non-sputum patients using BALF Mycobacterium culture + BALF-AFB smear + GeneXpert MTB/RIF + Gene chip as the gold standard, BALF-AFB smear showed low diagnostic performance, while, though GeneXpert MTB/RIF and gene chip had good diagnostic performance, combining GeneXpert MTB/RIF and gene chip improved the diagnostic value to a great extent. These findings might provide cues for the diagnosis improvement of pulmonary tuberculosis in suspected patients with negative sputum smear or no sputum.

Culture-negative, sputum smear-negative pulmonary TB represents about 30% to 60% of the patients with TB,^[10]^ indicating that many patients can present with no symptoms while still spread the disease.^[11,12]^ Even in patients with symptoms and signs, a failure to isolate *M. tuberculosis* from the appropriate samples cannot exclude the diagnosis of active TB.^[8,9]^ In patients with negative sputum smears, a clinical algorithm failed to fully diagnose the patients.^[14]^ In the present study, patients with symptoms were evaluated by BALF *Mycobacterium* culture, and 24 patients were diagnosed with TB. Then, 3 tests were performed for *M. tuberculosis* detection. All 3 tests had poor or moderate performance in diagnosing TB in such patients.

AFB smear microscopy should be performed in all patients suspected with TB.^[3,5]^ It is typically performed on sputum samples, with a reported sensitivity of about 70% by 3 AFB smears.^[3,5]^ Of note, AFB also indiscriminately identifies other *Mycobacterium* species as well as *M. tuberculosis*.^[3]^ The present study was performed in sputum smear-negative or sputum-negative patients. Therefore, AFB was performed on the BALF as suggested.^[3]^ Among the 3 tests, the results showed that BALF-AFB had the lowest diagnostic value for TB in these patients. Indeed, previous study suggested that BALF-AFB smear had a low AUC, with both sensitivity and PPV of 0%, indicating poor predictive value.^[15]^ Furthermore, the κ value between the BALF-AFB smear and the gold standard was negative, indicating that the results were the pure product of chance. Those results contradicted with previous studies indicating that bronchoscopy could provide adequate material for diagnosing TB in sputum smear-negative patients.^[16,17]^ A concurrent respiratory infection was reported to be associated with negative AFB.^[18]^ The presence of a pulmonary cavity, age, and a positive interferon-γ release assay could predict positive BALF-AFB in sputum smear-negative patients.^[19]^ Hence, several factors appear to influence the positivity of BALF-AFB. Those factors should be explored in further studies and considered in the analyses.

NAAT could increase the TB diagnostic yield.^[1,5]^ Previous studies showed that the diagnosis of smear-negative TB was greatly improved by GeneXpert MTB/RIF.^[20–26]^ Gene chip methods for detecting *M. tuberculosis* strains also showed good diagnostic values for sputum smear-negative TB.^[27,28]^ The strengths of NAATs include rapid testing within hours and distinguishing between *M. tuberculosis* and non-TB mycobacteria.^[5]^ In the present study, the combination of GeneXpert MTB/RIF and Gene chip for *Mycobacterium* strain identification yielded the highest AUC, but it was still lower than that in previous studies conducted in various populations of patients with TB.^[20–25]^ On the other hand, the GeneXpert MTB/RIF was shown to have a low sensitivity in patients with smear-negative TB.^[29,30]^ Previous studies also showed a relatively high diagnostic value for TB.^[27,31]^ Nevertheless, the low sensitivity of the GeneXpert MTB/RIF assay in the present study might be related to the selection of the comparator. A previous study suggested that the GeneXpert MTB/RIF assay and culture had similar results in sputum-negative patients,^[13]^ while another study showed higher performance of the GeneXpert MTB/RIF assay compared to culture in sputum.^[6]^ Hence, the selection of culture as the gold standard in this study might influence the results. However, the GeneXpert MTB/RIF assay still had undeniable advantages over culture due to its simultaneous detection of *M. tuberculosis* and rifampicin resistance, rapid testing of within 2 hours, and the involvement of minimal manipulations and hence minimal biohazard.^[7]^

The combination of the 2 genetic tests had the highest AUC and sensitivity for TB, as well as the highest specificity and accuracy. Indeed, there was discrepancy between the 2 tests. GeneXpert MTB/RIF test could be used for the automatic detection and drug susceptibility test of *M. tuberculosis*, but it could not effectively detect non-TB mycobacteria and TB with resistance to isoniazid.^[32,33]^ On the other hand, the gene chip performed better than the GeneXpert MTB/RIF test in detecting isoniazid-resistant *M. tuberculosis*.^[34]^ Therefore, the results of the 2 tests could also be inconsistent in the presence of specific *M. tuberculosis* strains. A combination of tests is probably the key to improving the diagnosis of TB in sputum smear-negative and non-sputum patients with suspected TB. Additional studies are warranted to determine such a combination. Of note, AFB still has its usefulness and should not be discarded in the diagnostic workup of a patient. Indeed, the GeneXpert MFB/Rif assay detected only *M. tuberculosis*. Hence, a negative GeneXpert MFB/Rif assay in the presence of a positive AFB indicated the presence of non-TB mycobacteria, which provided important information for the guidance of adequate treatments.^[7]^

The misdiagnosis of TB is relatively common and is influenced by 5 main factors, which could also be involved in the decreased AUC when combining the GeneXpert MTB/RIF and gene chip tests. First, a low-grade positivity was observed in patients with low loads of *M. tuberculosis*, especially in women and older adults.^[35]^ Second, in some laboratories, negative smear results could be due to improper sample handling, slide preparation, training, and slide reading.^[36,37]^ Third, cross-contamination should be considered.^[38]^ Forth, the simultaneous infection with other pathogens, including human immunodeficiency virus, increased the likelihood of negative smears.^[39,40]^ Fifth, imaging examinations often could not differentiate TB from other respiratory diseases with similar signs and symptoms.^[41]^ There are other methods for the detection of *M. tuberculosis*. An enzyme-linked immunospot showed good performance in BALF from smear-negative patients.^[42]^ New methods based on metagenome sequencing are also emerging.^[43]^ Such methods should also be explored to elaborate testing algorithms or test combinations to improve the diagnosis of TB.

*Mycobacterium* culture is the gold standard for diagnosing TB,^[5]^ which was suggested over 40 years ago as an appropriate technique in patients with sputum smear-negative results or incapable of producing sputum.^[44]^ However, it is still not a perfect test and that some patients could have been misdiagnosed in the present study.^[19,45]^

This study had limitations. It was a single-center study with a small sample size. No power analysis was performed. The laboratory tests were the 3 tests usually performed at the authors’ institutions, and additional tests were not evaluated. Prospective multicenter studies are needed to provide further high-level evidence.

### 4.1. Conclusions

In conclusion, for TB in sputum smear-negative and non-sputum patients using BALF Mycobacterium culture + BALF-AFB smear + GeneXpert MTB/RIF + Gene chip as the gold standard, BALF-AFB smear showed low diagnostic performance, while, though GeneXpert MTB/RIF and gene chip had good diagnostic performance, combining GeneXpert MTB/RIF and gene chip improved the diagnostic value to a great extent. These results call for additional studies, a combination of tests, or algorithms to improve the diagnosis of TB in sputum smear-negative and non-sputum patients.

## Author contributions

**Data curation:** Xiaopeng Cheng, Lerong Chen, Liangliang Wu, Jing Xin, Jianying Cai.

**Formal analysis:** Wenli Wan, Jianping Peng, Liangliang Wu, Jing Xin.

**Methodology:** Xiaopeng Cheng, Lerong Chen.

**Writing – original draft:** Xiaopeng Cheng, Lerong Chen, Jianying Cai.
